# Religious Service Attendance and Deaths Related to Drugs, Alcohol, and Suicide Among US Health Care Professionals

**DOI:** 10.1001/jamapsychiatry.2020.0175

**Published:** 2020-05-06

**Authors:** Ying Chen, Howard K. Koh, Ichiro Kawachi, Michael Botticelli, Tyler J. VanderWeele

**Affiliations:** 1 Human Flourishing Program, Harvard Institute for Quantitative Social Science, Cambridge, Massachusetts; 2 Harvard T. H. Chan School of Public Health, Department of Epidemiology, Boston, Massachusetts; 3 Harvard T. H. Chan School of Public Health, Department of Health Policy and Management, Boston, Massachusetts; 4 Harvard Kennedy School, Cambridge, Massachusetts; 5 Harvard T. H. Chan School of Public Health, Department of Social and Behavioral Sciences, Boston, Massachusetts; 6 Grayken Center for Addiction at Boston Medical Center, Boston, Massachusetts

## Abstract

**Importance:**

The increase in deaths related to drugs, alcohol, and suicide (referred to as *deaths from despair*) has been identified as a public health crisis. The antecedents associated with these deaths have, however, seldom been investigated empirically.

**Objective:**

To prospectively examine the association between religious service attendance and deaths from despair.

**Design, Setting, and Participants:**

This population-based cohort study used data extracted from self-reported questionnaires and medical records of 66 492 female registered nurses who participated in the Nurses’ Health Study II (NHSII) from 2001 through 2017 and 43 141 male health care professionals (eg, dentist, pharmacist, optometrist, osteopath, podiatrist, and veterinarian) who participated in the Health Professionals Follow-up Study (HPFS) from 1988 through 2014. Data on causes of death were obtained from death certificates and medical records. Data analysis was conducted from September 2, 2018, to July 14, 2019.

**Exposure:**

Religious service attendance was self-reported at study baseline in response to the question, “How often do you go to religious meetings or services?”

**Main Outcomes and Measures:**

Deaths from despair, defined specifically as deaths from suicide, unintentional poisoning by alcohol or drug overdose, and chronic liver diseases and cirrhosis. Cox proportional hazards regression models were used to estimate the hazard ratio (HR) of deaths from despair by religious service attendance at study baseline, with adjustment for baseline sociodemographic characteristics, lifestyle factors, psychological distress, medical history, and other aspects of social integration.

**Results:**

Among the 66 492 female participants in NHSII (mean [SD] age, 46.33 [4.66] years), 75 incident deaths from despair were identified (during 1 039 465 person-years of follow-up). Among the 43 141 male participants in HPFS (mean [SD] age, 55.12 [9.53] years), there were 306 incident deaths from despair (during 973 736 person-years of follow-up). In the fully adjusted models, compared with those who never attended religious services, participants who attended services at least once per week had a 68% lower hazard (HR, 0.32; 95% CI, 0.16-0.62) of death from despair in NHSII and a 33% lower hazard (HR, 0.67; 95% CI, 0.48-0.94) of death from despair in HPFS.

**Conclusions and Relevance:**

The findings suggest that religious service attendance is associated with a lower risk of death from despair among health care professionals. These results may be important in understanding trends in deaths from despair in the general population.

## Key Points

**Question:**

Is frequent religious service attendance associated with a lower risk of deaths related to drugs, alcohol, and suicide (referred to as *deaths from despair*) among US health care professionals?

**Findings:**

In this cohort study of 66 492 female registered nurses and 43 141 male health care professionals in the US, attendance at religious services at least once per week was associated with a 68% lower hazard of death from despair among women and a 33% lower hazard among men compared with never attendance.

**Meaning:**

The findings suggest that frequent attendance at religious services is associated with lower subsequent risk of deaths from despair.

## Teaser

This cohort study assesses the association of frequent religious service attendance with deaths related to drugs, alcohol, and suicide (referred to as *deaths from despair*) among US health care professionals.

## Introduction

Life expectancy in the US has decreased for 3 consecutive years since 2015.^1,2^ In particular, the mortality rates among middle-aged non-Hispanic white men and women aged 25 to 64 years increased by 5.2% between 1999 and 2016, with the differences particularly pronounced among individuals without a college degree.^3,4^ This increase in midlife mortality was largely associated with the increasing deaths from suicide, unintentional drug (eg, opioids) and alcohol poisoning, and alcohol-associated chronic liver disease and cirrhosis.^2^ These 3 causes of death have increased in parallel since 1999.^5^ Economists Case and Deaton^5^ introduced the term *deaths of despair* because these deaths may share a context of hopelessness and indifference toward living.^5,6^ As Deaton testified before the US Senate, suicide, drug misuse, and heavy drinking are all “plausible outcomes of processes that have cumulatively undermined meaning of life.”^7^

Deaths from despair-related causes have been present for centuries and across demographic groups.^8^ However, the exact mechanisms driving the recent increase in such deaths among middle-aged white persons in the working class remain unclear. It was hypothesized that growing economic insecurity (eg, increasing unemployment) has led these individuals, as the first generation in recent US history, to fear that they may not fare as well as their parents socially and economically.^5^ Such frustrated expectations might be aggravated by the weakening in traditional social support systems, leading to an increasing sense of hopelessness.^4,9^ One leading example of such a weakened support system is the decline in religious participation, including declining service attendance.^4^

Religion may be a social determinant of health and is associated with various aspects of health and well-being.^10,11,12,13^ Religious participation may promote health and well-being through strengthening social integration, encouraging healthy behaviors, and providing a sense of hope, meaning, and purpose in life.^14^ Previous evidence has suggested that the communal aspect of religion, namely service attendance, was inversely associated with various factors related to despair (eg, lower risk of suicidality, heavy drinking, substance misuse, and depression).^11,15,16^ Nevertheless, the extent to which service attendance is inversely associated with overall deaths from such conditions considering the composite deaths from despair remains unknown. To address this issue, we analyzed data from 2 prospective cohorts of US health professionals to examine the association between religious service attendance and deaths of potentially despair-associated conditions in men and women. Whereas the context of despair may vary across sociodemographic groups, the fundamental processes leading to despair (ie, loss of meaning in life) and the mechanisms whereby religious involvement helps counteract such processes may be similar across settings.^14,17^ Therefore, evidence from this study may help increase understanding of the recent trends in deaths from despair in the general population.

## Methods

### Study Population

This cohort study used data from the Nurses’ Health Study II (NHSII)^18^ from 2001 through June 30, 2017, and the Health Professionals Follow-Up Study (HPFS)^19^ from 1988, through January 31, 2014. The NHSII is an ongoing cohort of US female registered nurses, and the HPFS is an ongoing cohort of US male health care professionals. In both cohorts, participants have been followed up biennially with a response rate that exceeds 90% in each follow-up cycle. This study was approved by the institutional review board at the Brigham and Women’s Hospital. Participants’ return of a completed baseline questionnaire was considered to indicate informed consent.

We considered the NHSII 2001 supplementary survey and the HPFS 1988 survey in which religious service attendance was first assessed as the baseline for this study. Follow-up for mortality continued through June 30, 2017, in NHSII and January 31, 2014, in HPFS. Participants who did not respond to the baseline survey and those who died or had a diagnosis of cardiovascular disease or cancer before baseline were excluded from all analyses. Participants were followed up from return of the baseline questionnaire until death, loss to follow-up, or the end of follow-up, whichever came first. This yielded a sample of 66 492 participants in NHSII and 43 141 participants in HPFS (eMethods, eFigure 1, and eFigure 2 in the Supplement). Data analysis was conducted from September 2, 2018, to July 14, 2019.

### Measurements

In both cohorts, religious service attendance was assessed at study baseline with the question: “How often do you go to religious meetings or services?” Responses were grouped into 3 categories: never, less than once per week, and at least once per week.^15^

Deaths were identified from reports by next of kin, the National Death Index, state statistics records, and the postal system.^20^ Causes of death were identified by physicians by reviewing death certificates and medical records. We followed previous work^5^ in measuring deaths from despair. Specifically, the following codes of the *International Classification of Diseases, Eighth Revision (ICD-8)* were used in NHSII and the *International Classification of Diseases, Ninth Revision (ICD-9*) were used in HPFS: E950-E959 to define suicide; *ICD-8* E850 to E859, E860, E980, N965, N967, and N970 and *ICD-9* E850 to E859, E860, E980, E935, E937, and E939 to define unintentional poisoning by alcohol and overdose of prescription and illegal drugs; and 571 to define chronic liver diseases and cirrhosis.

Data on all covariates were obtained from self-reports of individuals at baseline. The following covariates were available in both cohorts: age, race/ethnicity, geographic region, living arrangement, employment status, preventive health care use, alcohol intake, smoking status, caffeine intake, body mass index, physical activity, and history of hypertension, hypercholesterolemia, and diabetes. To examine the role of service attendance independently from other aspects of social integration, we followed previous work^15^ to additionally adjust for a social integration score^21^ measuring major aspects of social integration other than service attendance (ie, an overall score was derived based on the following components: marital status, number of close friends, number of close relatives, frequency of contact with these social ties, and participation in other social groups), and quartiles of the score were created in both cohorts. In NHSII, we also adjusted for night-shift work schedule, household income, childhood abuse, menopausal status, menopausal hormone use, and depression. In HPFS, we additionally controlled for occupation, phobic anxiety symptoms, and history of kidney failure. Because depression was not assessed before service attendance in HPFS, we further adjusted for subsequent antidepressant use as a sensitivity analysis in this cohort (eMethods in the Supplement).

### Statistical Analysis

Cox proportional hazards regression models (with age in months as the time scale stratified by calendar time) were used to estimate hazard ratios (HRs) for deaths from despair by service attendance at baseline, adjusting for baseline sociodemographic characteristics, lifestyle factors, psychological distress, medical history, and other aspects of social integration. In HPFS, because depression was not measured before service attendance, we additionally adjusted for subsequent antidepressant use as a sensitivity analysis. Multiple imputation (with 5 imputed data sets) was performed to impute missing data on the exposure and all covariates.^22,23,24^

A number of additional sensitivity analyses were performed. First, to evaluate departures from the proportional hazards model assumption, we examined the interaction terms between age and service attendance and also analyzed the data using stratification on propensity score quintiles. Second, to reduce the concern that the association of service attendance with lower mortality, if any, was attributable to underlying impairment at baseline, we reanalyzed the models excluding participants who died of any cause during the first 3 years of follow-up. Third, to evaluate the potential bias from competing causes of death (ie, deaths from other causes may hinder the possibility that deaths from despair would have a chance to occur), a multinomial logistic regression model was used to examine service attendance in association with both deaths from despair and deaths from other causes. With rare events and minimal censoring, this strategy would approximate cause-specific cumulative incidence.^25^ Fourth, because approximately two-thirds of deaths from despair in this sample were suicides, we reanalyzed the models to examine service attendance in association with suicide and nonsuicide deaths (ie, combining deaths from poisoning and liver diseases) separately. Fifth, we calculated E-values^26,27^ to evaluate robustness of the results to potential unmeasured confounding.

All statistical analyses were performed using SAS, version 9.4 (SAS Institute Inc). All statistical tests were 2-sided, and *P* < .05 was considered to be statistically significant, although more precise *P* values and 95% CIs are given in all cases.

## Results

Among the 66 492 female participants in NHSII, the mean (SD) baseline age was 46.33 (4.66) years; among the 43 141 male participants in HPFS, the mean (SD) baseline age was 55.12 (9.53) years. In both cohorts, participants were predominantly non-Hispanic white, had relatively high socioeconomic status, and were generally healthy at baseline. Of the 2 cohorts, approximately 40% (NHSII: 44.1%; HPFS: 42.5%) of the participants reported attending religious services at least once per week, approximately 30% (NHSII: 31.3%; HPFS: 27.5%) attended services fewer than once per week, and 30% (NHSII: 24.6%; HPFS: 30.0%) reported never attendance. Compared with those attending services less frequently, participants who attended services at least once per week reported greater social integration, healthier behaviors, and less depression at baseline (Table 1).

**Table 1.** Age-Adjusted Participant Characteristics by Religious Service Attendance at Study Baseline^a^

Characteristic	Religious service attendance					
	NHSII 2001 (n = 66 419)			HPFS 1988 (n = 34 569)		
	Never	<1 Time/wk	≥1 Time/wk	Never	<1 Time/wk	≥1 Time/wk
Participants, No.	16 357	20 787	29 275	10 376	9499	14 694
Age, mean (SD), y^b^	47.1 (4.5)	46.1 (4.7)	46.1 (4.7)	55.5 (9.9)	54.7 (9.5)	55.3 (9.4)
Non-Hispanic white, %	95.4	94.8	95.6	95.5	95.5	97.0
Geographic region, %						
Northeast	32.6	35.7	31.9	29.4	18.0	18.3
Midwest	27.4	32.1	37.4	21.8	26.2	33.8
South	16.3	17.4	19.5	23.3	26.0	30.5
West	23.7	14.9	11.3	25.5	29.8	17.3
Household income, mean (SD), $						
<50 000	16.5	14.6	17.0	NA	NA	NA
50 000-74 999	27.8	26.3	28.5	NA	NA	NA
75 000-99 999	20.2	21.4	22.0	NA	NA	NA
≥100 000	35.4	37.7	32.6	NA	NA	NA
Occupation, %						
Dentist	NA	NA	NA	55.9	56.8	57.3
Pharmacist	NA	NA	NA	7.1	8.8	9.4
Optometrist	NA	NA	NA	6.8	8.2	7.1
Osteopath	NA	NA	NA	3.7	4.1	3.5
Podiatrist	NA	NA	NA	2.9	3.4	2.3
Veterinarian	NA	NA	NA	23.7	18.6	20.3
Currently employed, %	90.4	90.2	86.5	NA	NA	NA
Employment status, %						
Full-time	NA	NA	NA	81.4	84.5	83.5
Part-time	NA	NA	NA	6.9	5.9	5.6
Retired	NA	NA	NA	11.1	9.2	10.4
Disabled	NA	NA	NA	0.6	0.4	0.4
Night-shift work, %						
None	89.4	89.0	91.0	NA	NA	NA
1-9 mo	5.5	5.7	4.9	NA	NA	NA
10-19 mo	1.9	1.9	1.6	NA	NA	NA
≥20 mo	3.3	3.5	2.5	NA	NA	NA
Live alone, %	12.6	8.4	6.2	9.6	5.9	3.5
Social integration score, %						
Lowest quartile	23.3	10.7	3.9	20.2	8.9	4.6
2nd	42.7	34.8	23.4	43.4	34.3	22.2
3rd	23.2	37.1	49.9	21.8	34.4	42.9
Highest quartile	10.8	17.5	22.8	14.6	22.5	30.3
Childhood abuse, mean (SD), score^c^	1.9 (1.6)	1.8 (1.5)	1.7 (1.5)	NA	NA	NA
Routine physical examination, %	75.2	78.2	79.6	72.9	78.8	77.0
Alcohol intake, %						
0 g/d	32.5	33.8	47.5	16.7	16.8	31.0
0.1-9.9 g/d	48.5	52.1	44.0	36.0	45.0	37.1
10.0-29.9 g/d	16.0	12.4	7.6	29.7	27.4	23.2
≥30.0 g/d	3.0	1.7	0.9	17.6	10.8	8.7
Cigarette smoking, %						
Never	55.9	62.3	73.9	43.5	46.6	53.9
Former	29.7	27.7	21.6	44.6	44.8	39.8
Current, cigarettes/d						
1-14	6.4	5.2	2.7	3.1	2.8	2.2
15-24	5.4	3.4	1.4	4.2	3.1	2.4
≥25	2.6	1.4	0.4	4.6	2.8	1.7
Physical activity, %						
<3.0 METS	21.1	19.2	18.6	20.9	18.7	19.9
3.0-8.9 METS	19.3	20.4	21.8	21.4	21.4	24.2
9.0-17.9 METS	18.6	19.9	21.1	16.8	18.6	18.6
18.0-26.9 METS	12.7	13.2	13.9	13.0	13.8	13.1
≥27.0 METS	28.3	27.3	24.6	28.0	27.5	24.2
Body mass index categories, %^d^						
<20	7.8	7.1	7.3	1.5	1.1	1.3
20.0-24.9	43.5	43.9	45.1	47.8	46.8	45.5
25.0-29.9	25.8	26.5	26.7	43.1	44.6	45.8
30.0-34.9	12.5	12.9	12.2	6.5	6.6	6.4
≥35.0	10.4	9.7	8.9	1.1	0.9	1.0
Caffeine intake, %						
Bottom quintile	16.6	18.0	24.5	16.6	17.0	22.8
Second	17.9	19.7	21.9	17.9	20.2	20.9
Third	20.7	21.0	18.8	20.4	21.3	19.1
Fourth	22.6	20.7	17.7	21.9	20.9	19.3
Top quintile	22.3	20.7	17.2	23.2	20.5	17.9
Clinical characteristic, %						
Hypertension	10.2	10.2	9.2	19.9	20.7	19.6
Hypercholesterolemia	13.9	13.9	13.1	10.6	11.5	10.1
Diabetes	1.7	1.9	1.7	2.4	2.6	2.4
Kidney failure	NA	NA	NA	0.1	0.1	0.1
Postmenopausal status	16.5	16.2	16.3	NA	NA	NA
Hormone use	11.4	11.4	11.4	NA	NA	NA
High anxiety symptoms	NA	NA	NA	5.5	6.1	6.3
Depression	13.1	10.7	7.9	NA	NA	NA
Antidepressant use	NA	NA	NA	1.2	1.0	1.2

Abbreviations: HPFS, Health Professionals Follow-up Study; METS, metabolic equivalents; NA, not assessed or not applicable; NHSII, Nurses’ Health Study II.

^a^ Values are standardized to the age distribution of the study population. Values of polytomous variables may not sum to 100% because of rounding.

^b^ Value is not age adjusted. The age range was 36-56 years in NHSII and 40-80 years in HPFS.

^c^ Childhood abuse score assessing physical, emotional, and sexual abuse during childhood and adolescence was created, ranging from 0 to 5, with a higher score indicating more severe abuse.^28^

^d^ Calculated as weight in kilograms divided by height in meters squared.

In NHSII, 75 deaths from despair were identified during 1 039 465 person-years of follow-up, including 43 suicides, 20 deaths from poisoning, and 12 deaths from liver disease. During 973 736 person-years of follow-up in HPFS, 306 deaths from despair were identified, including 197 suicides, 6 deaths from poisoning, and 103 deaths from liver diseases. The incidence rate of the composite deaths from despair decreased monotonically with increasing service attendance (Table 2).

**Table 2.** Deaths From Despair in the Nurses’ Health Study II (2001-2017) and Health Professionals Follow-up Study (1988-2014)^a^

Religious service attendance	Participants, No.	Follow-up, person-years	Deaths from despair, No.	Incidence rate, events per 100 000 person-years
Nurses’ Health Study II (2001)				
Never or almost never	16 357	255 085	35	14
<1 Time/wk	20 787	324 804	24	7
≥1 Time/wk	29 275	458 439	16	3
Missing	73	1137	0	0
All participants	66 492	1 039 465	75	7
Health Professionals Follow-up Study (1988)				
Never or almost never	10 376	232 385	103	44
<1 Time/wk	9499	218 021	61	28
≥1 Time/wk	14 694	335 445	63	19
Missing	8572	187 885	79	42
All participants	43 141	973 736	306	31

^a^ Deaths related to drugs, alcohol, and suicide are referred to as deaths from despair.

There was a monotonic association between service attendance and lower hazards of deaths from despair in both cohorts. In the multivariable-adjusted proportional hazards analysis, compared with individuals who never attended services, participants who attended services at least once per week had a 68% lower hazard (HR, 0.32; 95% CI, 0.17-0.59) of deaths from despair in NHSII and a 37% lower hazard (HR, 0.63; 95% CI, 0.45-0.88) in HPFS (Table 3). The associations remained robust when other aspects of social integration were further adjusted (HR, 0.32; 95% CI, 0.16-0.62; *P* < .001 in NHSII; HR, 0.67; 95% CI, 0.48-0.94; *P* = .02 in HPFS). In the sensitivity analysis in HPFS that additionally controlled for subsequent antidepressant use, the association did not change (HR, 0.67; 95% CI, 0.48-0.94). We did not find evidence suggesting violations of the proportional hazards assumption (none of the interaction terms reached *P* < .05), and propensity score analyses yielded similar results (NHSII: HR, 0.30; 95% CI, 0.16-0.59; HPFS: HR, 0.53; 95% CI, 0.37-0.76). The sensitivity analyses excluding participants who died during the first 3 years of follow-up also yielded similar results comparing those attending services at least weekly with those never attending services (eTable 1 in the Supplement). Moreover, the multinomial logistic regression model suggested that service attendance was inversely associated with deaths from despair and had inverse or null associations with deaths from other causes (eTable 2 in the Supplement), indicating little evidence of bias from competing risks. That is, if service attendance was positively associated with deaths from other causes, individuals might die of other causes, precluding the opportunity for deaths from despair; thus, the inverse association between service attendance and deaths from despair might be spurious, but there was no such evidence.^29^

**Table 3.** Baseline Religious Service Attendance and Hazard Ratio of Deaths From Despair^a^

Study, analysis	Religious service attendance			
	Never or almost never	HR (95% CI)		*P* value for trend
		<1 Time/wk	≥1 Time/wk	
**NHSII, 2001-2007 (n = 66 492)**				
Age-adjusted	1[Reference]	0.55 (0.32-0.92)	0.26 (0.14-0.47)	<.001
Multivariable-adjusted^b^	1 [Reference]	0.65 (0.38-1.12)	0.32 (0.17-0.59)	<.001
Fully adjusted^c^	1 [Reference]	0.66 (0.38-1.14)	0.32 (0.16-0.62)	<.001
**HPFS, 1998-2014 (n = 43 141)**				
Age-adjusted	1 [Reference]	0.74 (0.51-1.08)	0.51 (0.37-0.70)	<.001
Multivariable-adjusted^b^	1 [Reference]	0.88 (0.60-1.30)	0.63 (0.45-0.88)	.01
Fully adjusted^c^	1 [Reference]	0.92 (0.63-1.35)	0.67 (0.48-0.94)	.02

Abbreviations: HPFS, Health Professionals Follow-up Study; HR, hazard ratio; NHSII, Nurses’ Health Study II.

^a^ Deaths related to drugs, alcohol, and suicide are referred to as deaths from despair. Multiple imputation was performed to impute missing data on religious service attendance and the covariates.

^b^ In both studies, the multivariable-adjusted model controlled for age (years), race/ethnicity (non-Hispanic white, other), geographic region (Northeast, Midwest, South, or West), living arrangement (live alone, other), past 2-year preventive health care use (yes or no), alcohol intake (0 g/d, 0.1-9.9 g/d, 10.0-29.9g/d, or ≥30.0 g/d), smoking status (never, former, current 1-14 cigarettes per day, current 15-24 cigarettes per day, or ≥25 cigarettes per day), caffeine intake (quintiles), body mass index (calculated as weight in kilograms divided by height in meters squared) (<20.0, 20.0-24.9, 25.0-29.9, 30.0-34.9, or ≥35.0), physical activity (<3.0, 3.0-8.9, 9.0-17.9, 18.0-26.9, or ≥27.0 metabolic equivalents), history of hypertension (yes or no), hypercholesterolemia (yes or no), and diabetes (yes or no). In NHSII, the model also adjusted for past 2-year night-shift work schedule (none, 1-9 months, 10-19 months, or ≥20 months), employment status (currently employed, not employed), household income (<$50 000, $50 000-$74 999, $75 000-$99 999, or≥$100 000), childhood abuse score (assessing physical, emotional, and sexual abuse during childhood and adolescence, ranging from 0 to 5, with a higher score indicating more severe abuse^28^), menopausal status (premenopausal or uncertain, postmenopausal), menopausal hormone use (yes or no), and depression (yes or no). In HPFS, the model also adjusted for occupation (dentist, pharmacist, optometrist, osteopath, podiatrist, or veterinarian), employment status (full-time, part-time, retired, or disabled), high phobic anxiety symptoms (yes or no), and history of kidney failure (yes or no).

^c^ In both NHSII and HPFS, the fully adjusted model further adjusted for other aspects of social integration (quartiles, assessed with a social integration score derived without religious service attendance).

When suicides and nonsuicide deaths (ie, from poisoning or liver diseases) were considered separately, there was a 75% lower hazard of suicide (HR, 0.25; 95% CI, 0.10-0.60) in NHSII and a 48% lower hazard (HR, 0.52; 95% CI, 0.34-0.82) in HPFS associated with attending services at least once per week vs never attendance. With nonsuicide deaths, there was also evidence for an inverse association with weekly attendance in NHSII (HR, 0.28; 95% CI, 0.09-0.90) but not in HPFS (HR, 1.04; 95% CI, 0.51-2.12) (eTable 3 in the Supplement).

Sensitivity analyses using E-values^26,27^ suggested that the observed associations were at least moderately robust to potential unmeasured confounding (eg, confounding by personality factors, such as conscientiousness). For instance, to explain HRs of 0.32 for deaths from despair in NHSII and 0.67 in HPFS, an unmeasured confounder associated with both increased likelihood of service attendance and decreased likelihood of deaths from despair by risk ratios of 5.70-fold (in NHSII) and 2.35-fold (in HPFS) each above the measured covariates could suffice, but weaker confounding could not. To shift the upper CI to include the null value, an unmeasured confounder associated with increased service attendance and decreased deaths from despair by risk ratios of 2.6-fold (in NHSII) and 1.3-fold (in HPFS) could suffice, but weaker confounding could not. For comparability, associations of the measured covariates with deaths from despair in both cohorts are provided in eTable 4 and eTable 5 in the Supplement).

## Discussion

Using longitudinal data from 2 prospective cohorts of US health care professionals, this study suggests that religious service attendance was associated with lower risk of deaths from despair among both men and women, accounting for a wide range of potential confounders (including other aspects of social integration). Findings of this study were congruent with previous evidence suggesting that religious service attendance was inversely associated with all-cause mortality and various factors associated with despair^11,15,16^; positively associated with psychosocial well-being outcomes, such as greater purpose in life^12,14^; and often more strongly associated with subsequent health compared with other aspects of social integration.^13,15^ Of interest, the magnitude of the association between service attendance and lower risk of deaths from despair was larger for women than for men in this study. These results are similar to previous research on religion and all-cause mortality.^11^ The different effect sizes may reflect potential sex differences in the social and emotional experiences during service attendance^30^ or in the extent of participation for those who report attending services weekly (eg, whether weekly attendance involves only an hour of participation or a more substantial part of the day).^10,11^ Alternatively, these differences may suggest stronger associations of service attendance with health outcomes in younger vs older populations (NHSII participants were younger than HPFS participants).

Factors that lead to despair are likely numerous and may vary across socioeconomic groups. The original context for the introduction of the term deaths of despair was that among white individuals in the working class who have lower educational attainment, for whom deteriorating job opportunities had led to financial difficulties and frustrated expectations.^3,4^ Such conditions, further compounded by weakened social support structures (eg, declining rates of marriage, child-rearing, and religious service attendance) and lack of strong social safety nets (eg, limited worker retraining programs, unequal access to health care), may be associated with recent increases in some risk behaviors (eg, opioid misuse) in this group.^4^ Although many of these factors may be contextually specific to this population, evidence of despair likely manifests across social strata. For those with higher educational attainment, despair may be associated with factors other than material deprivation. For instance, health care professionals have a suicide rate more than twice that of the general population,^31^ which may be partly associated with their chronic burnout at work.^32,33^ Regardless of the context, however, a completed suicide, in most cases, indicates marked despair. The fundamental processes leading to despair—a loss of meaning in life—may also be similar across settings.^17^

For some individuals, religious participation may serve as an important antidote and an asset for sustaining a sense of hope and meaning.^10^ Despite an increasing secular trend, religious participation remains common in the US. In 2018, 76% of American individuals reported a religious affiliation, 50% regarded religion as very important, and 32% attended religious services in the past week.^34^ Many religious traditions explicitly prohibit self-injury and substance use and promote holistic health as an integral part of expressing religious values. For example, in Christianity, the human body is perceived as a temple worthy of protection and care; in Judaism, the practice of Sabbath facilitates rest, renewal, and connection with God, family, and community; in Islam, alcohol and drugs are prohibited as intoxicants that cloud the mind and upset harmony in one’s community.^14^ Religion may also be associated with strengthened psychosocial resilience by fostering a sense of peace and positive outlook, promoting social connectedness, and encouraging engagement in prosocial activities.^14,35^ In the context of trauma, such resources may provide healthy stress-coping strategies and revive a sense of meaning in difficult times and thereby counteract various processes associated with despair.

### Strengths and Limitations

To our knowledge, this is the first study that empirically examined potential antecedents of the composite deaths from despair. With longitudinal data from 2 large cohorts, results of this study were robust to extensive confounding control, various modeling strategies, and potential unmeasured confounding, especially in NHSII.

This study is subject to certain limitations. First, all the participants were health care professionals with relatively high educational attainment. The context of despair in this group, therefore, may be different from that in some previous work.^5^ Nevertheless, the fundamental processes associated with despair and the mechanisms whereby religious service attendance is inversely associated with such processes may be similar across contexts. Second, there was no detailed information in these cohorts about specific causes of death from liver diseases (ie, whether it was alcohol related). Data from the US, however, note that on average, half of deaths from liver disease are associated with alcohol intake or hepatitis C; the latter is also associated with greater intravenous drug use.^36^ We also could not control for baseline drug use or liver diseases because of lack of data. Nevertheless, the results remained similar in the analysis excluding participants who died during the first 3 years of follow-up, and there was also evidence that the observed associations were at least moderately robust to potential unmeasured confounding. Next, we could not confirm whether the deaths in this study were preceded by long-term despair. Although these causes of death, in most cases, share a context of hopelessness, they may sometimes not be associated with despair (eg, genetic dispositions or impulsiveness). Next, this study examined only 1 aspect of religious participation, namely service attendance. As an avenue for social integration, service attendance provides an opportunity to connect individuals with a group that shares similar beliefs and values. The convergence of shared beliefs and enhanced social connection may be associated with health benefits. However, other aspects of religious involvement also merit investigation, especially for religious traditions that do not convene congregational meetings on a regular basis. For individuals without religious beliefs, other avenues of social integration may likewise be pursued. Although the magnitude of health associations may not be as substantial, other forms of social integration are also associated with health and well-being.^11,37,38^

## Conclusions

The findings suggest that religious service attendance is associated with a lower risk of death from despair among registered nurses and health care professionals. These results may be important in understanding trends in deaths from despair in the general population.

## Notes

Supplement. eMethods. Study Population and Covariates Assessment eFigure 1. Flow Diagram of Sample Derivation in Nurses’ Health Study II eFigure 2. Flow Diagram of Sample Derivation in Health Professionals Follow-up Study eTable 1. Baseline Religious Service Attendance and Hazard Ratio of Deaths of Despair, Excluding Participants Who Died During the First Three Years of Follow-up eTable 2. Baseline Religious Service Attendance and Odds Ratio of Deaths of Despair and Deaths From All Other Causes eTable 3. Baseline Religious Service Attendance and Hazard Ratio of Deaths From Suicide and Deaths From Other Despair-Related Causes eTable 4. The Associations Between Covariates and Deaths From Despair in The Fully-Adjusted Model in Nurses’ Health Study II eTable 5. The Associations Between Covariates and Deaths From Despair in The Fully-Adjusted Model in Health Professionals Follow-up Study eReferences.
