# Supplementary material for: Religious Service Attendance and Deaths Related to Drugs, Alcohol, and Suicide Among US Health Care Professionals
Source: JAMA Psychiatry. 2020 May 6;77(7):1–9. doi: 10.1001/jamapsychiatry.2020.0175 (PMC7203669; doi:10.1001/jamapsychiatry.2020.0175)
Supplement: Supplement. — eMethods. Study Population and Covariates Assessment eFigure 1. Flow Diagram of Sample Derivation in Nurses’ Health Study II eFigure 2. Flow Diagram of Sample Derivation in Health Professionals Follow-up Study eTable 1. Baseline Religious Service Attendance and Hazard Ratio of Deaths of Despair, Excluding Participants Who Died During the First Three Years of Follow-up eTable 2. Baseline Religious Service Attendance and Odds Ratio of Deaths of Despair and Deaths From All Other Causes eTable 3. Baseline Religious Service Attendance and Hazard Ratio of Deaths From Suicide and Deaths From Other Despair-Related Causes eTable 4. The Associations Between Covariates and Deaths From Despair in The Fully-Adjusted Model in Nurses’ Health Study II eTable 5. The Associations Between Covariates and Deaths From Despair in The Fully-Adjusted Model in Health Professionals Follow-up Study eReferences. [file jamapsychiatry-77-737-s001.pdf]

Supplementary Online Content

Chen Y, Koh HK, Kawachi I, Botticelli M, VanderWeele TJ. Religious Service Attendance and Deaths Related to Drugs, Alcohol, and Suicide Among US Health Care Professionals. *JAMA Psychiatry*. Published online May 6, 2020. doi:10.1001/jamapsychiatry.2020.0175

eMethods. Study Population and Covariates Assessment

eFigure 1. Flow Diagram of Sample Derivation in Nurses' Health Study Ⅱ.

eFigure 2. Flow Diagram of Sample Derivation in Health Professionals Follow-up Study.

eTable 1. Baseline Religious Service Attendance and Hazard Ratio of Deaths of Despair, Excluding Participants Who Died During the First Three Years of Follow-up

eTable 2. Baseline Religious Service Attendance and Odds Ratio of Deaths of Despair and Deaths From All Other Causes

eTable 3. Baseline Religious Service Attendance and Hazard Ratio of Deaths From Suicide and Deaths From Other Despair-Related Causes

eTable 4. The Associations Between Covariates and Deaths from Despair in The Fully-Adjusted Model in Nurses' Health Study Ⅱ

eTable 5. The Associations Between Covariates and Deaths from Despair in The Fully-Adjusted Model in Health Professionals Follow-up Study

eReferences.

This supplementary material has been provided by the authors to give readers additional information about their work.

© 2020 Chen Y et al. *JAMA Psychiatry.*

eMethods. Study Population and Covariates Assessment

Study Population

The Nurses' Health Study II (NHSII) was established in 1989 and enrolled 116,429 U.S. female registered nurses aged 25 to 42 years.[1] The Health Professionals Follow-Up Study (HPFS) was initiated in 1986, which enrolled 51,529 U.S. male health professionals aged between 40 and 75 years.[2] In both cohorts, participants have been followed-up biennially with self-administered questionnaires, and the response rate exceeds 90% in each follow-up cycle.

Religious service attendance was first assessed in the NHSII 2001 Trauma Exposure and Post-traumatic Stress supplementary survey (N=67,905) and the HPFS 1988 survey (N=48,767). We considered these years as the baseline for this study. Follow-up for mortality continued through June 30, 2017 in NHSII and Jan 31, 2014 in HPFS. We excluded participants who died or had a diagnosis of cardiovascular disease or cancer before the study baseline (n=1,413 in NHSII, n=5,626 in HPFS). This yielded analytic samples of 66,492 participants in NHSII (75 cases of deaths of despair, over 1,039,465 person-years of follow-up), and 43,141 participants (306 cases of deaths of despair, over 973,736 person-years of follow-up) in HPFS (efigures 1 and 2).

Covariates Assessment

In HPFS, we controlled for baseline age (in years), race/ethnicity (researchers defined categories: non-Hispanic white, others), occupation (dentist, hospital pharmacist, optometrist, osteopath, pharmacist, podiatrist, veterinarian), geographic region (Northeast, South, West, Midwest), employment status (full time, part time, retired, disabled), living alone (yes, no), social integration (measured with the simplified Berkman-Syme Social Network Index,[3] derived excluding religious service attendance), alcohol consumption (0, 0.1 to 9.9, 10.0 to 29.9, 30.0+

© 2020 Chen Y et al. *JAMA Psychiatry.*

grams/day), smoking status (never smoker, former smoker, current smoker 1-14, 15-24, >=25 cigarettes/day), caffeine intake (mg/day; quintiles), physical activity (metabolic equivalent hours per week; quintiles), routine physical exam in the past 2 years (yes, no), BMI (<20.0, 20.0-24.9, 25.0-29.9, 30.0-34.9, >=35.0 kg/m^2), high phobic anxiety symptoms (yes, no), and history of hypertension (yes, no), hypercholesterolemia (yes, no), diabetes (yes, no), and renal failure (yes, no). Because depression was not measured prior to service attendance, we also controlled for subsequent anti-depressant use (yes, no) as a sensitivity analysis.

In NHSII, we controlled for baseline age (in years), race/ethnicity (researchers defined categories: non-Hispanic white, others), geographic region (Northeast, South, West, Midwest), household income (<$50 000, $50 000-$74 999, $75 000-$99 999, ≥$100 000), census tract median income (<$50 000, $50 000-$74 999, $75 000-$99 999, ≥$100 000), census tract college education rate (continuous), employment status (currently employed, non-employed), past 2-year rotating night shift work (0,1-9, 10-19, 20+ months), living alone (yes, no), childhood abuse (continuous score 0-5), social integration (measured with the simplified Berkman-Syme Social Network Index,[3] derived excluding religious service attendance), alcohol consumption (0, 0.1 to 9.9, 10.0 to 29.9, 30.0+ grams/day), smoking status (never smoker, former smoker, current smoker 1-14, 15-24, >=25 cigarettes/day), caffeine intake (mg/day; quintiles), physical activity (metabolic equivalent hours per week; quintiles), routine physical exam in the past 2 years (yes, no), BMI (<20.0, 20.0-24.9, 25.0-29.9, 30.0-34.9, >=35.0 kg/m^2), depression (yes, no), menopausal status (premenopausal or uncertain, postmenopausal), current hormone use (yes, no), and history of hypertension (yes, no), hypercholesterolemia (yes, no) and diabetes (yes, no).

© 2020 Chen Y et al. *JAMA Psychiatry.*

eFigure 1. Flow Diagram of Sample Derivation in Nurses' Health Study II

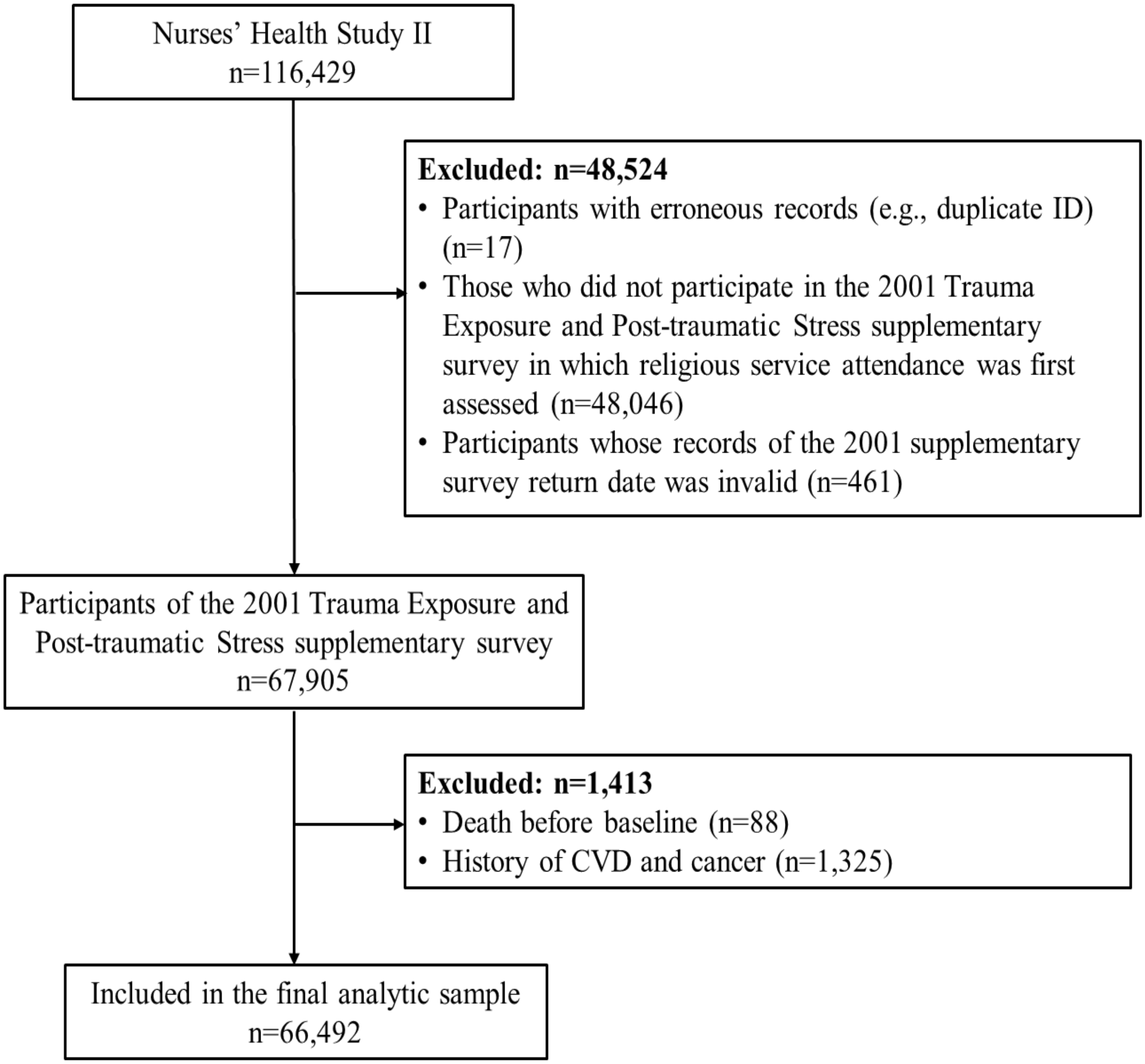

© 2020 Chen Y et al. *JAMA Psychiatry.*

eFigure 2. Flow Diagram of Sample Derivation in Health Professionals Follow-up Study

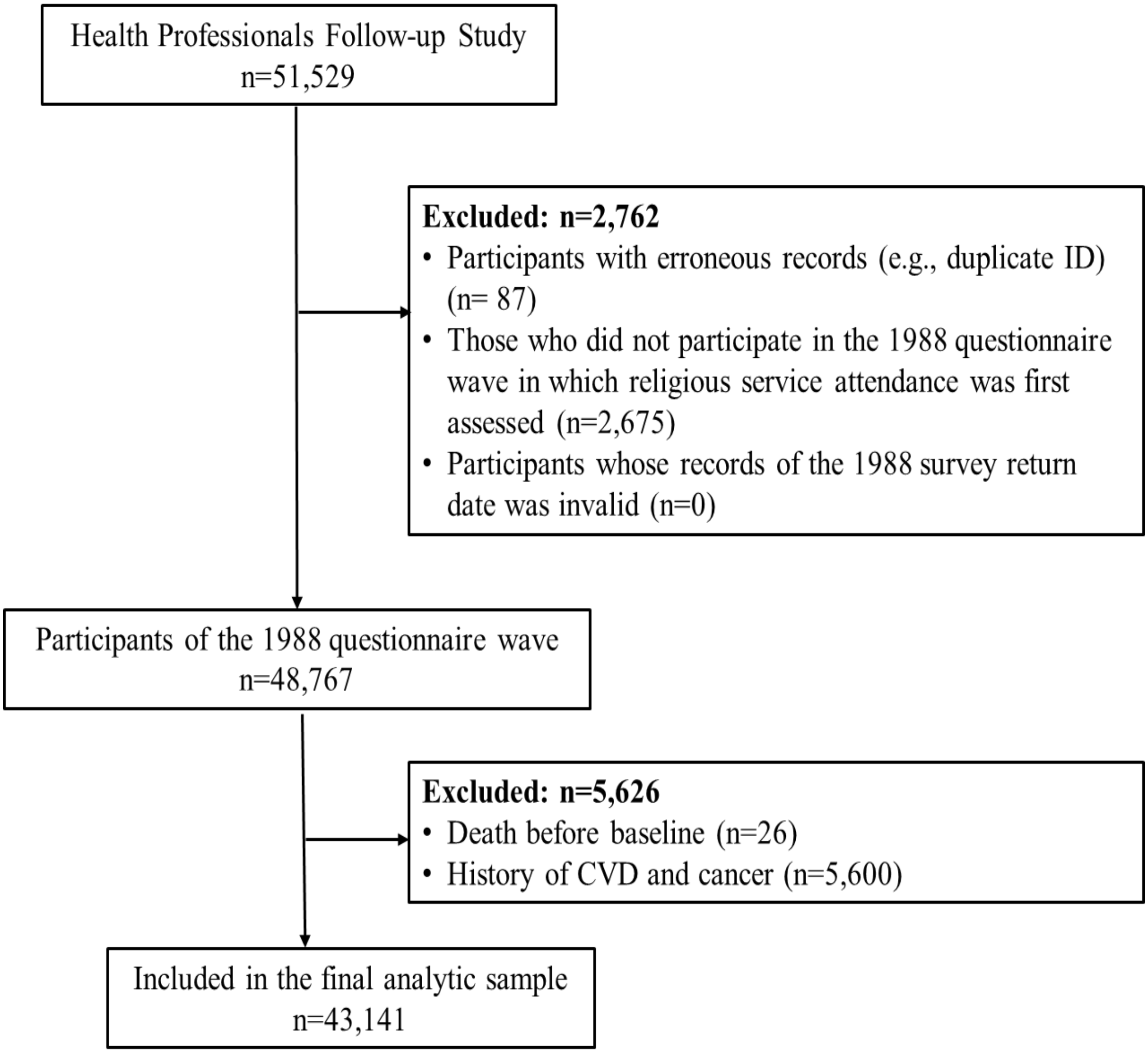

© 2020 Chen Y et al. *JAMA Psychiatry.*

eTable 1. Baseline Religious Service Attendance and Hazard Ratio of Deaths of Despair, Excluding Participants Who Died During the First Three Years of Follow-up (Nurses' Health Study II, 2001-2017 (N=66,318); Health Professionals Follow-up Study, 1988-2014 (N=42,499)[a]

	Religious Service Attendance					
	Never/almost never		Less than once/week		At least once/week	
	(reference)		HR (95% CI)	*P* value	HR (95% CI)	*P* value
NHSII						
Age-adjusted	1.00		0.60 (0.34, 1.05)	0.075	0.30 (0.16, 0.57)	<.001
Multivariable-adjusted [b]	1.00		0.69 (0.38, 1.24)	0.212	0.34 (0.18, 0.67)	0.002
Fully-adjusted [c]	1.00		0.70 (0.38, 1.27)	0.242	0.35 (0.17, 0.71)	0.004
HPFS						
Age-adjusted	1.00		0.76 (0.49, 1.16)	0.190	0.51 (0.36, 0.73)	<.001
Multivariable-adjusted [b]	1.00		0.90 (0.58, 1.41)	0.630	0.63 (0.43, 0.92)	0.019
Fully-adjusted [c]	1.00		0.95 (0.61, 1.46)	0.791	0.68 (0.46, 1.00)	0.050

Abbreviations: HR, hazard ratio; CI, confidence interval.

[a] Participants (n=174 in NHSII, 642 in HPFS) who died during the first 3 years of follow-up were removed. Multiple imputation was performed to impute missing data on religious service attendance and the covariates.

[b] In both NHSII and HPFS, the multivariable-adjusted model controlled for age (years), race/ethnicity (non-Hispanic white, others), geographic region (Northeast, Midwest, South, West), living arrangement (live alone, others), past 2-year preventive healthcare use (yes, no), alcohol intake (0, 0.1-9.9, 10.0-29.9, 30+ g/d), smoking status (never, former, current 1-14/d, current 15-24/day, >=25/day), caffeine intake (quintiles), body mass index (<20, 20-24.9, 25-29.9, 30-34.9, 35+ kg/m2), physical activity (<3, 3-8.9, 9-17.9, 18-26.9, ≥27 METS), history of hypertension (yes, no), hypercholesterolemia (yes, no), and diabetes (yes, no). In NHSII, the model also additionally adjusted for past 2-year night shift work schedule (none, 1-9 months, 10-19 months, 20+ months), employment status (currently employed, not employed), household income (<$50,000, $50,000-$74,999, $75,000-$99,999, ≥$100,000), childhood abuse (continuous score 0-5), menopausal status (postmenopausal, premenopausal or uncertain), menopausal hormone use (yes, no), and depression (yes, no), In HPFS, the model also additionally adjusted for occupation (dentist, pharmacist, optometrist, osteopath, podiatrist, veterinarian), employment status (full-time, part-time, retired, disabled), phobic anxiety (yes, no), and history of renal failure (yes, no).

[c] In both NHSII and HPFS, the fully-adjusted model further adjusted for other aspects of social integration (quartiles, assessed with a social integration score derived without religious service attendance).

© 2020 Chen Y et al. *JAMA Psychiatry.*

eTable 2. Baseline Religious Service Attendance and Odds Ratio of Deaths of Despair and Deaths from All Other Causes (Nurses' Health Study II, 2001-2017 (N=66,492); Health Professionals Follow-up Study, 1988-2014 (N=43,141)[a]

	Religious Service Attendance [b]				
	Never/almost never	Less than once/week		At least once/week	
	(reference)	OR (95% CI)	*P* value	OR (95% CI)	*P* value
NHSII (non-death as reference)					
Deaths of despair (n=75)	1.00	0.64 (0.37, 1.10)	0.109	0.32 (0.16, 0.63)	<.001
Deaths from other causes (n=1306)	1.00	0.96 (0.83, 1.11)	0.539	0.87 (0.74, 1.01)	0.068
HPFS (non-death as reference)					
Deaths of despair (n=306)	1.00	0.94 (0.65, 1.34)	0.709	0.66 (0.48, 0.93)	0.017
Deaths from other causes (n=19,198)	1.00	1.00 (0.93, 1.08)	0.966	0.95 (0.88, 1.02)	0.147

Abbreviations: OR, odds ratio; CI, confidence interval.

[a] A multinomial logistic regression model was used to examine deaths (non-deaths, deaths of despair, deaths from other causes) at the end of follow-up by baseline religious service attendance. Multiple imputation was used to impute missing data on religious service attendance and the covariates.

[b] In both cohorts, all models controlled for age (years), race/ethnicity (non-Hispanic white, others), geographic region (Northeast, Midwest, South, West), living arrangement (live alone, others), past 2-year preventive healthcare use (yes, no), alcohol intake (0, 0.1-9.9, 10.0-29.9, 30+ g/d), smoking status (never, former, current 1-14/d, current 15-24/day, >=25/day), caffeine intake (quintiles), body mass index (<20, 20-24.9, 25-29.9, 30-34.9, 35+ kg/m2), physical activity (<3, 3-8.9, 9-17.9, 18-26.9, ≥27 METS), history of hypertension (yes, no), hypercholesterolemia (yes, no) and diabetes (yes, no), and other aspects of social integration (quartiles, assessed with a social integration score derived without religious service attendance). In NHSII, all models also additionally adjusted for past 2-year night shift work schedule (none, 1-9 months, 10-19 months, 20+ months), employment status (currently employed, not employed), household income (<$50,000, $50,000-$74,999, $75,000-$99,999, ≥$100,000), childhood abuse (continuous score 0-5), menopausal status (postmenopausal, premenopausal or uncertain), menopausal hormone use (yes, no), and depression (yes, no), In HPFS, all models also additionally adjusted for occupation (dentist, pharmacist, optometrist, osteopath, podiatrist, veterinarian), employment status (full-time, part-time, retired, disabled), phobic anxiety (yes, no), and history of renal failure (yes, no).

© 2020 Chen Y et al. *JAMA Psychiatry.*

eTable 3. Baseline Religious Service Attendance and Hazard Ratio of Deaths From Suicide and Deaths From Other Despair-Related Causes (Nurses' Health Study II, 2001-2017 (N=66,492); Health Professionals Follow-up Study, 1988-2014 (N=43,141)[a]

	Religious Service Attendance				
	Never/almost never (reference)	Less than once/week		At least once/week	
		HR (95% CI)	*P* value	HR (95% CI)	*P* value
NHSII					
**Deaths from suicide**					
Age-adjusted	1.00	0.36 (0.18, 0.75)	0.006	0.21 (0.10, 0.46)	<.001
Multivariable-adjusted [b]	1.00	0.43 (0.21, 0.92)	0.029	0.26 (0.11, 0.59)	0.001
Fully-adjusted [c]	1.00	0.42 (0.20, 0.91)	0.028	0.25 (0.10, 0.60)	0.002
**Deaths from poisoning or liver diseases**					
Age-adjusted	1.00	0.44 (0.12, 1.59)	0.186	0.24 (0.09, 0.62)	0.004
Multivariable-adjusted [b]	1.00	0.52 (0.14, 1.87)	0.289	0.29 (0.10, 0.82)	0.020
Fully-adjusted [c]	1.00	0.51 (0.13, 2.01)	0.306	0.28 (0.09, 0.90)	0.034
HPFS					
**Deaths from suicide**					
Age-adjusted	1.00	0.75 (0.52, 1.09)	0.128	0.43 (0.28, 0.64)	<.001
Multivariable-adjusted [b]	1.00	0.86 (0.59, 1.27)	0.448	0.49 (0.32, 0.75)	0.001
Fully-adjusted [c]	1.00	0.90 (0.61, 1.33)	0.601	0.52 (0.34, 0.82)	0.005
**Deaths from poisoning or liver diseases**					
Age-adjusted	1.00	0.73 (0.35, 1.52)	0.383	0.67 (0.35, 1.31)	0.227
Multivariable-adjusted [b]	1.00	0.91 (0.41, 2.02)	0.814	0.97 (0.46, 2.02)	0.921
Fully-adjusted [c]	1.00	0.96 (0.45, 2.02)	0.904	1.04 (0.51, 2.12)	0.914

Abbreviations: HR, hazard ratio; CI, confidence interval.

[a] Multiple imputation was performed to impute missing data on religious service attendance and the covariates.
[b] In both NHSII and HPFS, the multivariable-adjusted model controlled for age (years), race/ethnicity (non-Hispanic white, others), geographic region (Northeast, Midwest, South, West), living arrangement (live alone, others), past 2-year preventive healthcare use (yes, no), alcohol intake (0, 0.1-9.9, 10.0-29.9, 30+ g/d), smoking status (never, former, current 1-14/d, current 15-24/day, >=25/day), caffeine intake (quintiles), body mass index (<20, 20-24.9, 25-29.9, 30-34.9, 35+ kg/m2), physical activity (<3, 3-8.9, 9-17.9, 18-26.9, ≥27 METS), history of hypertension (yes, no), hypercholesterolemia (yes, no), and diabetes (yes, no). In NHSII, the model also additionally adjusted for past 2-year night shift work schedule (none, 1-9 months, 10-19 months, 20+ months), employment status (currently employed, not employed), household income (<$50,000, $50,000-$74,999, $75,000-$99,999, ≥$100,000), childhood abuse (continuous score 0-5), menopausal status (postmenopausal, premenopausal or uncertain), menopausal hormone use (yes, no), and depression (yes, no), In HPFS, the model also additionally adjusted for occupation (dentist, pharmacist, optometrist, osteopath, podiatrist, veterinarian), employment status (full-time, part-time, retired, disabled), phobic anxiety (yes, no), and history of renal failure (yes, no).
[c] In both NHSII and HPFS, the fully-adjusted model further adjusted for other aspects of social integration (quartiles, assessed with a social integration score derived without religious service attendance).

© 2020 Chen Y et al. *JAMA Psychiatry.*

eTable 4. The Associations Between Covariates and Deaths From Despair in The Fully-Adjusted Model in Nurses' Health Study II (NHSII 2001-2017, N=66,492)[a]

	**Deaths from despair**	
	HR (95% CI)	*P* value
Religious service attendance		
- Never/almost never (ref)	1.00	
- Less than once/week	0.66 (0.38, 1.14)	0.13
- At least once/week	0.32 (0.16, 0.62)	<0.001
Non-Hispanic white (no as ref)	0.82 (0.32, 2.10)	0.68
Geographic region		
- Northeast (ref)	1.00	
- Midwest	1.37 (0.69, 2.72)	0.36
- South	2.38 (1.21, 4.67)	0.01
- West	2.21 (1.09, 4.49)	0.03
Household income		
- <$50,000 (ref)	1.00	
- $50,000-$74,999	0.65 (0.32, 1.31)	0.23
- $75,000-$99,999	1.00 (0.50, 2.01)	1.00
- >=$100,000	0.60 (0.27, 1.33)	0.21
Currently employed (no as ref)	0.38 (0.22, 0.67)	<0.001
Night shift work		
- none (ref)	1.00	
- 1-9 months	0.76 (0.23, 2.49)	0.65
- 10-19 months	2.06 (0.62, 6.88)	0.24
- 20+ months	3.02 (1.26, 7.23)	0.01
Live alone (no as ref)	1.60 (0.79, 3.27)	0.19
Social integration score		
- lowest (ref)	1.00	
- 2nd	0.88 (0.45, 1.73)	0.71
- 3rd	0.97 (0.43, 2.23)	0.95
- highest	0.92 (0.34, 2.50)	0.87
Childhood abuse (range: 1-5)	1.16 (1.00, 1.35)	0.05
Routine physical exam (no as ref)	0.56 (0.34, 0.92)	0.02
Alcohol intake		
- 0 g/d (ref)	1.00	
- 0.1-9.9 g/d	0.59 (0.31, 1.12)	0.11
- 10.0-29.9 g/d	0.77 (0.29, 2.01)	0.58
- 30+ g/d	1.93 (0.70, 5.30)	0.20
Cigarette smoking		
- never (ref)	1.00	
- former	1.73 (1.02, 2.94)	<0.05
- current 1-14/d	1.66 (0.63, 4.41)	0.31
- current 15-24/day	0.97 (0.28, 3.33)	0.96
- current >=25/day	1.79 (0.49, 6.49)	0.38
Physical activity		
- <3 METS (ref)	1.00	
- 3-8.9 METS	0.59 (0.27, 1.31)	0.19
- 9-17.9 METS	1.13 (0.57, 2.24)	0.70

© 2020 Chen Y et al. *JAMA Psychiatry.*

- 18-26.9 METS	0.28 (0.08, 0.98)	0.05
- >=27 METS	1.00 (0.52, 1.93)	1.00
BMI categories		
- <20 (ref)	1.00	
- 20-24.9	0.67 (0.31, 1.44)	0.31
- 25-29.9	0.55 (0.23, 1.32)	0.18
- 30-34.9	0.87 (0.36, 2.09)	0.75
- 35+	0.38 (0.12, 1.18)	0.09
Caffeine intake		
- bottom quintile (ref)	1.00	
- 2nd	1.44 (0.66, 3.13)	0.36
- 3rd	1.17 (0.54, 2.55)	0.69
- 4th	1.02 (0.44, 2.37)	0.96
- top quintile	1.03 (0.46, 2.33)	0.94
Hypertension (no as ref)	1.56 (0.76, 3.18)	0.22
Hypercholesterolemia (no as ref)	0.70 (0.33, 1.47)	0.34
Diabetes (no as ref)	1.17 (0.27, 5.17)	0.83
Post menopause status (no as ref)	2.22 (0.88, 5.60)	0.09
Hormone use (no as ref)	1.00 (0.37, 2.69)	1.00
Depression (no as ref)	2.19 (1.22, 3.92)	<0.01

[a] Multiple imputation was performed to impute missing data on religious service attendance and the covariates. Cox proportional hazard model was used to examine the hazards of deaths from despair by religious service attendance in the fully-adjusted model.

© 2020 Chen Y et al. *JAMA Psychiatry.*

eTable 5. The Associations Between Covariates and Deaths From Despair in The Fully-Adjusted Model in Health Professionals Follow-up Study (HPFS 1988-2014, N=43,141)[a]

	**Deaths from despair**	
	HR (95% CI)	*P* value
Religious service attendance		
- Never/almost never (ref)	1.00	
- Less than once/week	0.92 (0.63, 1.35)	0.67
- At least once/week	0.67 (0.48, 0.94)	0.02
Non-Hispanic white (no as ref)	2.04 (0.89, 4.69)	0.09
Geographic region		
- Northeast (ref)	1.00	
- Midwest	0.81 (0.58, 1.15)	0.24
- South	1.08 (0.78, 1.48)	0.65
- West	0.80 (0.56, 1.14)	0.21
Occupation		
- Dentist (ref)	1.00	
- Pharmacist	0.56 (0.33, 0.96)	0.03
- Optometrist	0.52 (0.29, 0.95)	0.03
- Osteopath	0.82 (0.44, 1.53)	0.53
- Podiatrist	1.84 (1.08, 3.13)	0.03
- Veterinarian	1.12 (0.84, 1.50)	0.43
Employment status		
- Full-time (ref)	1.00	
- Part-time	0.88 (0.51, 1.50)	0.63
- Retired	1.35 (0.89, 2.05)	0.16
- Disabled	0.92 (0.22, 3.88)	0.91
Live alone (no as ref)	2.17 (1.51, 3.11)	<0.001
Social integration score		
- lowest (ref)	1.00	
- 2nd	0.87 (0.59, 1.29)	0.49
- 3rd	0.72 (0.45, 1.16)	0.17
- highest	0.79 (0.52, 1.21)	0.28
Routine physical exam (no as ref)	0.99 (0.73, 1.33)	0.93
Alcohol intake		
- 0 g/d (ref)	1.00	
- 0.1-9.9 g/d	0.71 (0.50, 1.00)	0.05
- 10.0-29.9 g/d	1.00 (0.70, 1.41)	0.99
- 30+ g/d	2.06 (1.43, 2.96)	<0.001
Cigarette smoking		
- never (ref)	1.00	
- former	1.04 (0.78, 1.38)	0.80
- current 1-14/d	1.22 (0.64, 2.32)	0.55
- current 15-24/day	1.36 (0.72, 2.55)	0.34
- current >=25/day	2.95 (1.84, 4.74)	<0.001
Physical activity		
- <3 METS (ref)	1.00	
- 3-8.9 METS	0.90 (0.65, 1.26)	0.55
- 9-17.9 METS	0.83 (0.58, 1.20)	0.32

© 2020 Chen Y et al. *JAMA Psychiatry.*

- 18-26.9 METS	0.65 (0.42, 1.00)	0.05
- >=27 METS	0.71 (0.50, 1.01)	0.05
BMI categories		
- <20 (ref)	1.00	
- 20-24.9	1.24 (0.52, 2.93)	0.63
- 25-29.9	0.79 (0.61, 1.03)	0.08
- 30-34.9	1.24 (0.81, 1.88)	0.32
- 35+	3.33 (1.68, 6.60)	<0.001
Caffeine intake		
- bottom quintile (ref)	1.00	
- 2nd	1.01 (0.70, 1.46)	0.94
- 3rd	1.02 (0.71, 1.46)	0.93
- 4th	0.78 (0.54, 1.14)	0.20
- top quintile	0.68 (0.46, 1.01)	0.06
Hypertension (no as ref)	1.38 (1.06, 1.81)	0.02
Hypercholesterolemia (no as ref)	1.08 (0.76, 1.54)	0.67
Diabetes (no as ref)	1.41 (0.75, 2.65)	0.28
Renal failure (no as ref)	6.97 (1.57, 31.04)	0.01
High anxiety symptoms (no as ref)	0.75 (0.42, 1.34)	0.33

[a] Multiple imputation was performed to impute missing data on religious service attendance and the covariates. Cox proportional hazard model was used to examine the hazards of deaths from despair by religious service attendance in the fully-adjusted model.

© 2020 Chen Y et al. *JAMA Psychiatry.*

eReferences

1. Solomon CG, Willett WC, Carey VJ, et al. A prospective study of pregravid determinants of gestational diabetes mellitus. *JAMA.* 1997;278(13):1078-1083.
2. Rimm EB, Giovannucci EL, Willett WC, et al. Prospective study of alcohol consumption and risk of coronary disease in men. *The Lancet.* 1991;338(8765):464-468.
3. Berkman LF, Syme SL. Social networks, host resistance, and mortality: a nine-year follow-up study of Alameda County residents. *Am J Epidemiol.* 1979;109(2):186-204.

© 2020 Chen Y et al. *JAMA Psychiatry.*
